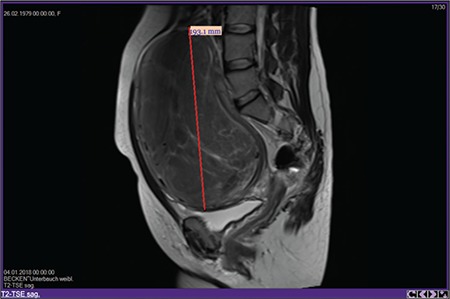# Laparoscopic assisted robotic myomectomy of a huge myoma; Does robotic surgery change the borders in minimally invasive gynecology?

**DOI:** 10.4274/jtgga.galenos.2019.2019.0029

**Published:** 2019-08-28

**Authors:** Özgüç Takmaz, Savaş Gündoğan, Esra Özbaşlı, Emine Karabük, Murat Naki, Faruk Köse, Mete Güngör

**Affiliations:** 1 Clinic of Obstetrics and Gynecology, Acıbadem Mehmet Ali Aydınlar University, Maslak Hospital, İstanbul, Turkey; 2 Clinic of Obstetrics and Gynecology, Acıbadem Mehmet Ali Aydınlar University, Atakent Hospital, İstanbul, Turkey

**Keywords:** Robotic myomectomy, huge myoma, fibroid

## Abstract

Today, the adoption of minimal invasive gynecologic procedures is expanding their routine use in clinical practice. Until recently, a diameter of 8 cm was the recommended maximal size for laparoscopic removal of fibroids. However, robot-assisted laparoscopy improved the capacity and the feasibility of the many gynecologic procedures. Here, we report a video of robotic myomectomy of a huge myoma.

## Introduction

To demonstrate the feasibility of robotic myomectomy of a huge fibroid, we recorded a robotic myomectomy operation video of a 19 cm diameter (FIGO type 3-4) myoma (Canadian Task Force Classification III) at a university-affiliated private hospital.

A 38-year-old, gravida 2 (vaginal birth) patient with a 19 cm intramural fibroid was admitted to our clinic with a request of endoscopic removal of the fibroid. The patient was given detailed information about risk of the surgery, defining the risk of disseminating malignant cells through the abdominal cavity. It was then decided to perform a myomectomy operation using a robotic platform. The operation was performed using a Da Vinci Xi platform (Intuitive Surgical, Inc., Sunnyvale, Ca); the patient card was docked centrally, and three robotic arms and an assistant port with a smoke evacuator (AirsealR SurgiQuest, Inc., CT, USA) were used.

The surgical time (skin to skin) was 205 min, and the docking time was 6 min. A 2.0 barbed suture was used for uterine closure. The estimated blood loss (calculated with the difference between irrigation and suction) was 350 cc, and two erythrocyte suspension transfusions were given after the operation. The first gas discharge was 13 hours after the surgery, the length of hospital stay was 2 days. No complications occurred peri-operatively.

Huge fibroids can be removed using robot-assisted laparoscopy.

**Video 1. DOI: 10.4274/jtgga.galenos.2019.2019.0029.video.1**

## Figures and Tables

**Figure:**
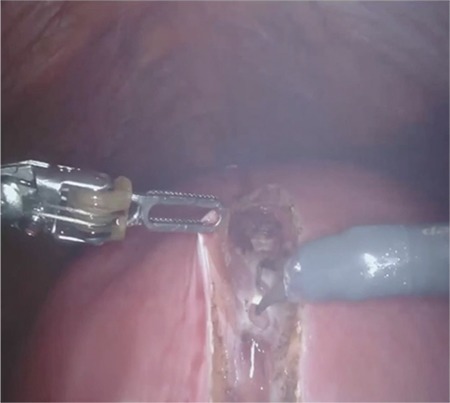


**Figure:**